# Superficial Calcification With Rotund Shape Is Associated With Carotid Plaque Rupture: An Optical Coherence Tomography Study

**DOI:** 10.3389/fneur.2020.563334

**Published:** 2020-09-18

**Authors:** Xuan Shi, Yunfei Han, Min Li, Qin Yin, Rui Liu, Fang Wang, Xiaohui Xu, Yunyun Xiong, Ruidong Ye, Xinfeng Liu

**Affiliations:** ^1^Department of Neurology, Affiliated Jinling Hospital, Medical School of Nanjing University, Nanjing, China; ^2^Department of Neurology, Jiangsu Province Hospital of Chinese Medicine, Nanjing University of Chinese Medicine, Nanjing, China; ^3^China National Clinical Research Center for Neurological Diseases, Beijing, China; ^4^Vascular Neurology, Department of Neurology, Beijing Tiantan Hospital, Capital Medical University, Beijing, China

**Keywords:** calcification, atherosclerosis, carotid stenosis, optical coherence tomography (OCT), plaque rupture

## Abstract

**Background:** Plaque rupture is an important etiology for symptomatic carotid stenosis. The role of calcification in the plaque vulnerability has been controversial. We aimed to detect the geometric features of calcifications in carotid plaque and to examine its association with plaque rupture.

**Methods:** Optical coherence tomography assessment of carotid plaque was performed in 88 patients. Calcification shape was evaluated through quantitative measurements of the long and short axis, area size, circumference, calcification arc, and longitudinal length. Calcification location was analyzed through the distance to the lumen. Furthermore, we developed idealized fluid-structure interaction models to investigate the association of calcification shape and plaque stress.

**Results:** A total of 33 ruptured plaques and 30 non-ruptured plaques were recognized. Ruptured plaques had more multiple calcifications and protruded calcifications. The calcifications in the ruptured plaques displayed a remarkably lower long-axis/short-axis (L/S) ratio than in the non-ruptured plaques (*p* = 0.001). We classified calcification shape into crescentic calcification (L/S > 2.5) and rotund calcification (L/S ≤ 2.5). Rotund-shaped calcifications were more common in ruptured plaques than in non-ruptured plaques (*p* = 0.02). Superficial calcifications with minimal distance to the lumen ≤ 50 μm accounted for 79.4% of all calcifications in the ruptured plaques, and only 7.7% in the non-ruptured plaques (*p* < 0.001). Biomechanical analysis showed that the plaque with rotund-shaped calcification developed 7.91-fold higher von Mises stress than the plaque with crescentic calcification.

**Conclusions:** Superficial calcifications and rotund-shaped calcifications are associated with carotid plaque rupture, suggesting that calcification location and shape may play a key role in plaque vulnerability.

## Introduction

Carotid atherosclerosis remains an important cause of stroke and transient ischemic attack. Ruptured plaques, defined as lesions with a structural defect in the fibrous cap with the underlying necrotic core, are often associated with ulcerated plaques on carotid angiography (1). Disruption of the fibrous cap exposes the highly thrombogenic core to the blood, and then leads to thrombus formation and subsequent embolization in carotid artery (2). Plaque rupture is thus a critical pathogenetic process for symptomatic carotid stenosis (3). The role of differential atherosclerotic compositions in the plaque stability remains controversial. Calcification is a common plaque component with uncertain effects in the atherosclerosis progression. Calcified plaque has traditionally been regarded as stable atheroma. Asymptomatic plaques have higher degree of calcification than symptomatic plaques (4). In contrast, small calcifications may represent a dynamic inflammation-stimulated process, which has been associated with accelerated disease progression and greater atheroma burden (5–7). Several studies have reported that spotty (8) and multiple calcifications (9) occurred more often in vulnerable plaques.

Optical coherence tomography (OCT) has lately been developed as a high-resolution imaging modality which provides maximum axial resolution of 15–20 μm, thus offering a unique tool to image the detailed structure of arterial plaque *in vivo*. Several studies have utilized OCT to investigate lipid-rich component, thrombus and fibrous cap thickness in the carotid artery plaques (10–12). But the role of calcification in the carotid atherosclerotic vulnerability has rarely been investigated. In this context, the present study aimed to detect a detailed geometric feature of calcification in the carotid plaque and to examine its association with plaque rupture.

## Materials and Methods

### Study Population

From April 2013 to November 2018, 88 consecutive patients with 90 cervical internal carotid artery (ICA) stenosis who evaluated by OCT at Department of Neurology, Jinling Hospital were enrolled. The application of OCT to human cerebral angiography was approved by the Ethics Committee of Jinling Hospital. Informed consent was obtained from all patients before digital subtraction angiography (DSA) and OCT imaging. Twenty-seven lesions in 26 patients were excluded for the following reasons: incomplete target lesions imaging (*n* = 13), poor image quality (*n* = 12) and non-atherosclerotic etiologies (*n* = 2). Finally, 63 lesions in 62 patients were enrolled in the final analysis.

### Clinical Data Collection

Demographic and clinical data were collected. Clinical information including age, sex, body mass index, history of hypertension, diabetes mellitus, hyperlipidemia, coronary heart disease, current history of smoking, and current medication was collected. Ipsilateral lesions with stroke or transient ischemic attack (TIA) lesion were defined as a documented history of transient ischemic attack, amaurosis fugax, or stroke in the vascular territory supplied by the ICA within 6 months (10). TIA was defined as an episode of temporary and focal cerebral dysfunction of vascular origin, lasting for a maximum 24 h, leaving no persistent neurologic deficits. Amaurosis fugax was defined as a sudden loss of vision of presumed vascular origin and confined to one eye (13). Ischemic stroke was defined as a symptomatic neurologic deterioration lasting at least 24 h that was not attribute to a non-ischemic cause, or a new symptomatic neurologic deterioration that was not attributable to a non-ischemic cause and was accompanied by neuroimaging evidence of a new brain infarction (14). Levels for triglycerides, total cholesterol, high-density lipoprotein, low-density lipoprotein, creatinine, glucose were also recorded.

### OCT Image Acquisition

A DynaCT angiography scanner (Siemens Axiom Artis dTA, Siemens Healthcare, Erlangen, Germany) was used for DSA examination. The grade of carotid stenosis was evaluated by using angiography according to North America Symptomatic Carotid Endarterectomy trial (NASCET) criteria (15). Frequency domain OCT systems (ILUMEN OPTIS System or C7-XR, St. Jude Medical, Abbott Vascular, USA) were used in all cases. Carotid OCT images were acquired using a 2.7-F Dragonfly OCT imaging catheter (C7 Dragonfly Catheter or Dragonfly Duo Catheter, St. Jude Medical, Abbott Vascular, USA). An 8-F guide catheter was placed in the distal of carotid artery C1 segment. OCT catheter was inserted through an 8-F sheath over the 0.014-inch PT guide wire, then navigated past the ICA lesion. Image acquisition was obtained when blood was removed by injection of 20 ml undiluted iodixanol 320 (GE Healthcare Ireland Limited, County Cork, Ireland) at the flow rate of 10 ml/s through the guiding catheter. As images completed the Z-offset adjustment, then automatic pullbacks were withdrawn by an electronic control covering 54 mm of the vessel at a velocity of 20 or 25 mm/s. All images were stored digitally for subsequent offline analysis using proprietary software (ILUMEN OPTIS System, St. Jude Medical, Abbott Vascular, USA). The evaluation and treatment were under the auspices of an interventional neurologist with expensive carotid OCT experience (Y.R.), and two interventional neurologists (H.Y. and L.M.).

### OCT Image Analysis

All images were analyzed by 2 independent investigators (XS and RL) who were blinded to the clinical presentations and angiographic findings. To determine the reproducibility of OCT qualitative assessments, data were analyzed by 2 independent analysts (XS and RL) and repeated 1 month after initial analysis by the same analyst (XS). Images were considered non-analyzable if there was a serious artifact, or if intraluminal blood impaired the assessment of a continuous 270° arc (10).

Plaque rupture was identified by the presence of fibrous cap discontinuity with a clear cavity formed inside the plaque (Supplementary Figure). Rupture sites separated by a length of artery containing smooth lumen contours and no cavity were considered as different plaque ruptures (11). Lipid plaque was semi-quantified identified when the arc of lipid was >90° in any of the images within a plaque (16). Thin-cap fibroatheroma (TCFA) was identified as lipid-rich plaques with a minimum fibrous cap thickness measuring <65 μm. Fibrous cap thickness was measured at the thinnest part 3 times, and the average value was calculated. Thrombus was defined as a backscattering protrusion into the carotid lumen with signal-intensity-free shadowing (16). Macrophage accumulations were recognized as signal-rich, distinct, or confluent punctate regions that exceed the intensity of background speckle noise (16). Neovascularization was defined as signal absent holes within a plaque measuring between 50 and 300 μm in diameter and visible on at least 3 consecutive frames on pullback imaging (16). Cholesterol crystals were identified as thin linear structures with high reflectivity and low signal attenuation (16).

The minimum lumen area (MLA) and minimum lumen diameter (MLD) of each segment was measured, as well as reference vessel area (RVA) and reference vessel diameter (RVD). References were defined as the most “normal-appearing” segment distal to the lesions on the basis of OCT-derived NASCET criteria (10, 16). Percent area stenosis was calculated as ([distal reference vessel area - MLA] / distal reference vessel area) × 100%. Percent diameter stenosis was calculated as ([distal reference vessel diameter - MLD]/distal reference vessel diameter) × 100%.

### Quantitative Assessment of Calcification

**Figure 1:**
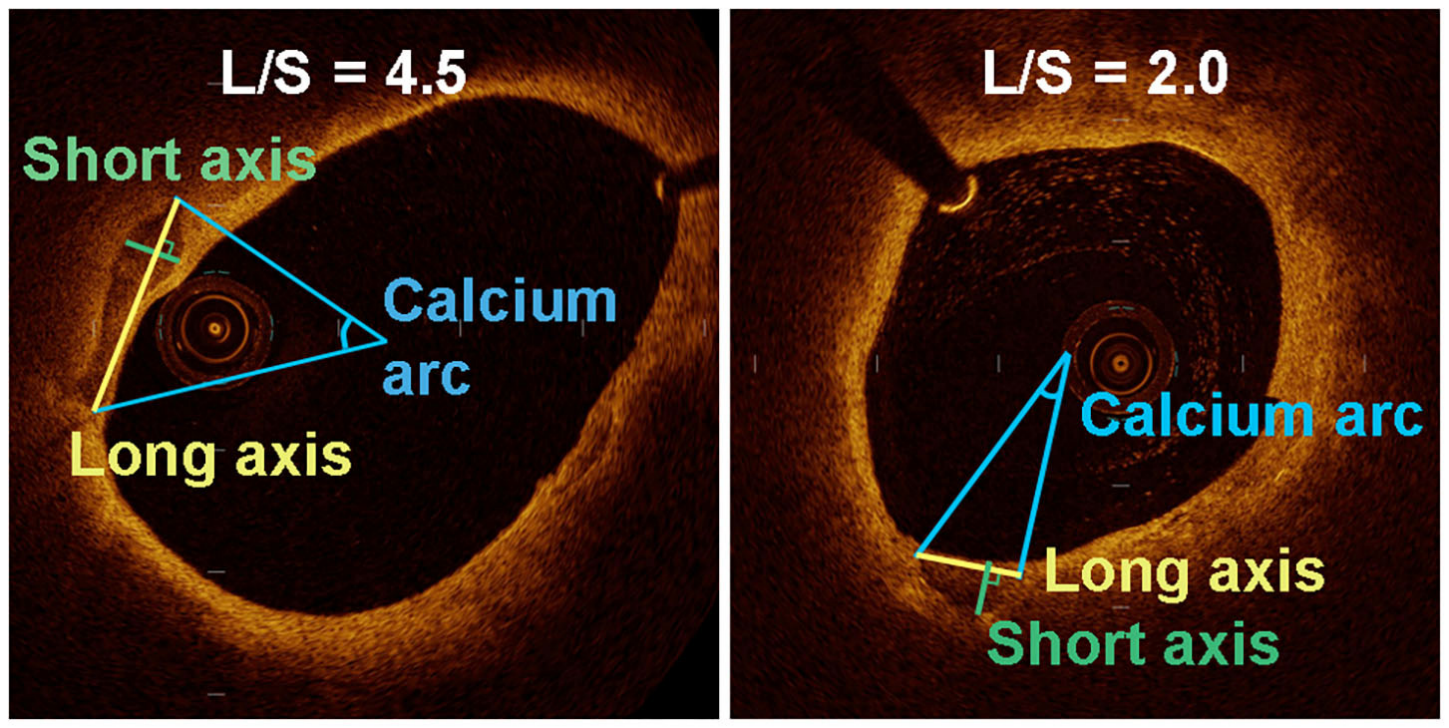
Quantitative analysis of various parameters for the assessment of long axis and short axis in a transverse section of an OCT-image of two plaques with different calcification shape.

**Figure 2:**
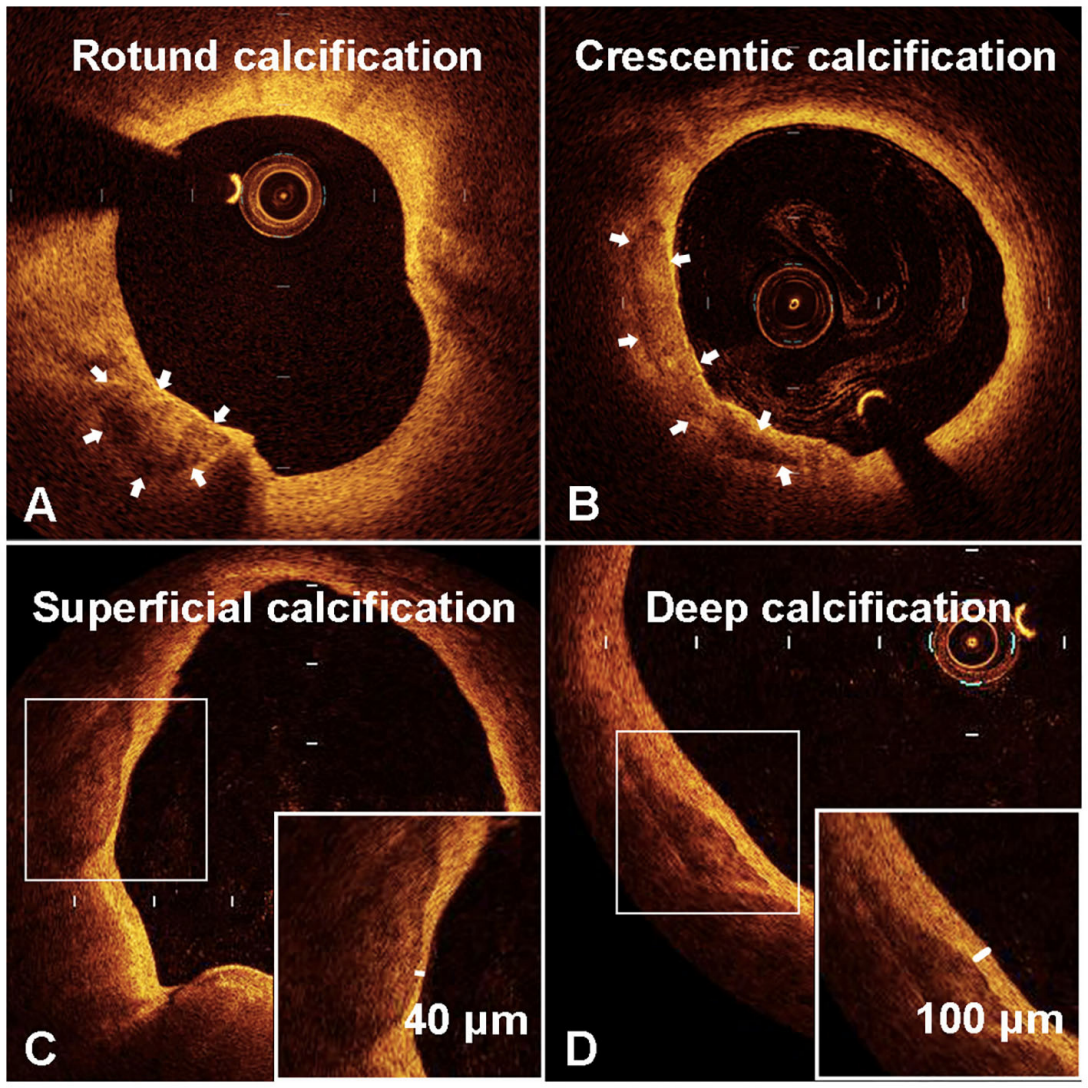
Representative optical coherence tomography (OCT) images of rotund calcification **(A)**, crescentic calcification **(B)**, superficial calcification **(C)**, and deep calcification **(D)**.

Calcification was recognized as well-delineated, low signal attenuation with sharp borders (16). We measured the long axis (L) and short axis (S) of the calcifications on the cross-sectional OCT image with maximum calcification area (Figure 1). To this end, the lumen center and maximum calcification arc were first determined by the OCT software. The line connecting two tangency points of maximum arc on the calcification semilune was defined as L. The longest line segment perpendicular to L inside the calcification was defined as S. We then calculated the long-axis/short-axis (L/S) ratio of each calcification. Based on L/S, we classified calcification shape into crescentic calcification (L/S > 2.5) and rotund calcification (L/S ≤ 2.5) (Figure 2).

The minimal depth between the inner edge of calcification and the luminal surface was measured 3 times, and the average value was calculated. Length of calcification was calculated by the total slice number multiplied by the frame interval. The angles of each calcification were analyzed every 1 mm using the center of the mass of the lumen. Maximum calcification arc was defined as the widest angle in which calcifications were detectable, and mean calcification arc was defined as total calcification angle divide for the number of frames presenting with calcification (17). Calcification area size and circumference were measured following the sharply delineated contour of each calcification on the maximum area frame. The surface circumference/area ratio of each calcification was also calculated. The calcification index was calculated as the product of calcification length and mean arc for each calcification (17). Spotty calcifications were defined to be those with length <4 mm and maximal arc <90°, and deposits not meeting these criteria were classified as large calcifications (18). Protruded calcification were considered as calcified mass into the lumen with or without eruption (19). The minimum longitudinal distance from the nearest edge of each calcification to the MLA site was also measured.

### Biomechanical Analysis

We constructed the two idealized models with our defined types of calcification morphology by three-dimensional, two-way fluid-structure interaction (FSI) computational model in Unigraphics NX 12.0 (Siemens, German) and the finite element program ANSYS Workbench 19.2 (ANSY, America). We assessed stress on a plaque that comprises three main tissue types: calcification, fibrous and a blunt cresent-shaped lipid core. In this model, the lumen and vessel diameters were chosen to be 3 and 10 mm, respectively, and the vessel wall thickness was 1.5 mm. The lumen was modeled with an eccentricity of 2 mm with respect to arterial center. The lipid core in the maximum cross-section slice was constructed by extending a 140° crescent with thickness of 2.3 mm. The idealized models only differed in calcification morphology. The minimum depth between the inner edge of calcification and lumen was 0.1 mm.

Both the artery wall and plaque components were assumed to be hyper-elastic, isotropic, incompressible, and heterogeneous. The following parameters were used in a plane-stress model: Young modulus (*E*) in circumferential (θ) and radial (*r*) directions, ν_*rθ*_ and ν_*rz*_that are the Poisson ratios in *r-*θ and θ*-z* planes, respectively, as well as *G*_*rθ*_ that is the shear modulus in *r-*θ plane. The material constitution and corresponding parameters of all tissues were adapted from previous studies (20, 21): artery, *E*_*r*_ = 10 kPa, *E*_θ_ = 100 kPa, *G*_*rθ*_ = 50 kPa, ν_*rθ*_ = 0.01, ν_*rz*_ = 0.27; fibrous tissue, *E*_*r*_ = 50 kPa, *E*_θ_ = 1,000 kPa, *G*_*rθ*_ = 500 kPa, ν_*rθ*_= 0.01, ν_*rz*_ = 0.27; lipid, *E*_*r*_ = 1 kPa, *E*_θ_ = 1 kPa, *G*_*rθ*_ = 1 kPa, ν_*rθ*_ = 0.01, ν_*rz*_ = 0.27; calcium, *E*_*r*_ = 9452.7 kPa, *E*_θ_ = 9500.2 kPa, *G*_*rθ*_ = 9475.2 kPa, ν_*rθ*_ = 0.01, ν_*rz*_ = 0.27. Blood flow was assumed to be laminar, Newtonian, viscous, and incompressible. The material properties for blood was as follow: density = 1,050 kg/m^3^, viscosity = 3.50E-03 Pa/s.

For the vessel wall, both the inlet and outlet were restricted from moving radially while free to move in the axial direction. Inner surfaces of the arterial wall which come in contact with the fluid domain were marked as FSI interfaces. The solid model was meshed using 10-node tetrahedrons: 261,319 nodes and 175,223 elements for the model with rotund calcification, 254,609 nodes and 171,570 elements for the model with crescentic calcification, respectively. The fluid model was meshed using 8-node hexahedrons and luminal blood flow domain of 5,115 nodes and 9,000 elements. All FSI simulations were performed on high-performance computing cluster composed of four quad core Xeon CPU with a shared memory of 32 GB RAM.

### Statistical Analysis

Continuous variables with normal distribution were described as mean ± SD, and variables with skewed distribution were described as median (the 25th to the 75th percentile). The distribution of continuous variables was tested by the Kolgormonov-Smirnov test. Comparisons for continuous variables were performed using an independent samples *t*-test or Mann–Whitney *U*-test as appropriate. Categorical variables were summarized as frequencies and percentages, and comparisons were performed using a Fisher exact test. Univariate and multivariable logistic regression analyses were performed to identify potential risk factors for the presence of plaque rupture. Inter-observer and intra-observer reliability was assessed using Cohen Kappa test for categorical variables and intra-class correlation test for continuous variables. All tests were 2-sided, and *p*-values below a threshold of 0.05 were considered significant. All statistical analyses were performed using IBM SPSS software 23.0 (IBM, Armonk, NY). Receiver-operating characteristic curve (ROC) analysis was performed to assess the best cut-off value of L/S and depth of each calcification to predict plaque rupture using MedCalc Version 18.2.1 (MedCalc Software, Ostend, Belgium).

## Results

### Patient Characteristics

A total of 62 patients were enrolled in this study, including 32 patients presented with ruptured plaques and 30 patients with non-ruptured plaques. Ruptured plaques were more found in patients with ipsilateral stroke/TIA (*p* = 0.03). There were no significant differences in age, clinical and biochemical parameters between patients with or without plaque rupture (Table 1).

**Table 1:** Patient characteristics.

	**Patients with ruptured plaques (*n* = 32)**	**Patients with non-ruptured plaques (*n* = 30)**	***P*-value**
Age, y	67 (62–69)	63 (58–70)	0.50
Male sex, *n* (%)	27 (84.38)	21 (70.00)	0.13
Body mass index, kg/m^2^	24.90 ± 3.12	24.54 ± 2.41	0.63
Clinical features, *n* (%)			
Ipsilateral stroke/TIA	15 (46.88)	6 (20.00)	0.03
Hypertension	26 (81.25)	27 (90.00)	1.00
Diabetes mellitus	13 (40.63)	11 (36.67)	0.80
Coronary heart disease	12 (37.50)	7 (23.33)	0.27
Current smoking	16 (50.00)	10 (33.33)	0.20
Current medication, *n* (%)			
Antiplatelet therapy	25 (78.13)	28 (93.33)	0.26
Statin	27 (84.38)	29 (96.67)	0.20
Antihypertensive medication	20 (62.50)	22 (73.33)	0.36
Antidiabetic medication	13 (40.63)	13 (43.33)	0.83
Biochemical parameters			
Triglycerides, mmol/L	1.33 (1.04–1.91)	1.13 (0.91–1.38)	0.12
Total cholesterol, mmol/L	3.53 ± 0.94	3.64 ± 1.03	0.66
HDL, mmol/L	0.99 ± 0.20	1.04 ± 0.32	0.48
LDL, mmol/L	1.72 (1.54–2.32)	2.03 (1.53–2.41)	0.74
Creatinine, μmol/L	63.00 (55.00–72.00)	65.00 (51.40–71.00)	0.95
Serum glucose, mmol/L	5.40 (4.70–6.90)	5.60 (5.10–6.20)	0.70

*HDL, high-density lipoprotein; LDL, low-density lipoprotein; TIA, transient ischemic attack*.

### Plaque Characteristics

A total of 33 ruptured plaques and 30 non-ruptured plaques were recognized from 62 patients (Supplementary Table I). Ruptured plaques in carotid artery had smaller minimal luminal area compared with non-ruptured plaques (*p* = 0.04). Nevertheless, the area stenosis of lesions with ruptured plaques were similar with lesions with non-ruptured plaques (*p* = 0.26).

The minimal fibrous cap thickness was markedly smaller in ruptured plaques than in non-ruptured plaques (*p* < 0.001). 75.8% of the ruptured plaques presented with TCFA, as a presence of fibrous cap <65 μm illustrated by OCT. By contrast, only 23.3% of the non-ruptured plaques contained TCFA. Lipid-rich plaque was more common in the ruptured plaques than in non-ruptured plaques (81.8 vs. 40.0% *p* = 0.001). The incidence of thrombus (27.3 vs. 0.0%, *p* = 0.002) and neovascularization (18.2 vs. 0.0%, *p* = 0.02) were likewise significantly higher in ruptured plaques. Macrophages and cholesterol crystals appeared similarly in plaques with or without rupture.

We further classified plaques into mild stenosis lesions (<50%) and severe stenosis lesions (≥50%). Lesions with severe stenosis were more presented with lipid-rich plaque, overlying thinner fibrous cap, than lesions with mild stenosis (81.8 vs. 44.2%, *p* = 0.04). However, the incidence of plaque disruption, thrombus, macrophage accumulations and cholesterol crystals appeared similarly in lesions with mild or severe stenosis (Supplementary Table II).

### Calcification Analysis

A total of 47 calcifications were identified in 63 plaques by OCT imaging. Among them, 34 calcifications presented in 15 ruptured plaques and 13 calcifications presented in 9 non-ruptured plaques (Table 2). There was no difference in the presence of calcium deposits between ruptured and non-ruptured plaques (45.5 vs. 30.0%, *p* = 0.30). However, multiple calcifications were more common in ruptured plaques than in non-ruptured plaques (33.3 vs. 10.0%, *p* = 0.04; Supplementary Table I).

**Table 2:** Calcification characteristics in ruptured and non-ruptured plaques.

	**Calcifications in ruptured plaques (*n* = 34)**	**Calcifications in non-ruptured plaques (*n* = 13)**	***p*-value**
L/S	2.76 (1.61–4.02)	5.03 (3.40–7.03)	0.001
Rotund calcification, *n* (%)	16 (47.06)	1 (7.69)	0.02
Crescentic calcification, *n* (%)	18 (52.94)	12 (92.31)	0.02
Minimal distance to the lumen, mm	0.04 (0.03–0.05)	0.17 (0.07–0.21)	<0.001
Superficial calcification, *n* (%)	27 (79.41)	1 (7.69)	<0.001
Deep calcification, *n* (%)	7 (20.59)	12 (92.31)	<0.001
Calcification area, mm^2^	1.43 (0.48–2.55)	1.05 (0.60–2.10)	0.81
Calcification surface, mm	4.67 ± 2.61	6.43 ± 2.84	0.05
Surface circumference/area, mm/mm^2^	3.92 (2.78–5.74)	6.16 (3.40–8.59)	0.04
Calcification length, mm	2.31 ± 1.53	2.02 ± 1.63	0.56
Maximum calcification arc, °	47.45 (35.63–72.38)	48.10 (31.50–71.95)	0.67
Mean calcification arc, °	38.90 (30.50–62.54)	36.63 (27.93–53.09)	0.81
Calcification index, °mm	81.48 (38.31–129.34)	57.84 (25.48–193.28)	0.35
Distance to the MLA site, mm	1.10 (0.08–2.55)	2.65 (1.25–3.94)	0.04
Spotty calcification, *n* (%)	29 (85.29)	11 (84.62)	1.00
Protruded calcification, *n* (%)	16 (47.06)	0 (0.00)	0.002

*L/S, long axis/short axis ratio; MLA, minimum luminal area*.

We measured the long axis (L) and short axis (S) of the semilunar-shaped calcifications in the axial OCT images (Figure 1). The calcifications in the ruptured plaques displayed a remarkably lower L/S ratio compared with calcifications in the non-ruptured plaques (2.76 vs. 5.03, *p* = 0.001; Table 2), indicating calcification shape may be associated with carotid plaque rupture. The potential cut-off values of L/S ratio predicting plaque rupture were sequentially measured by ROC analysis (Supplementary Table IV). We thus defined calcifications with L/S ≤ 2.5 as *rotund calcifications*, whereas calcifications with L/S > 2.5 were defined as *crescentic calcifications* (AUC, 0.81; 95% CI, 0.67–0.94; *p* = 0.001). 47.1% of the calcifications in the ruptured plaques were rotund-shaped calcifications. Meanwhile only 7.7% of the calcifications in the non-ruptured plaques were rotund-shaped calcifications (*p* = 0.02).

**Figure 3:**
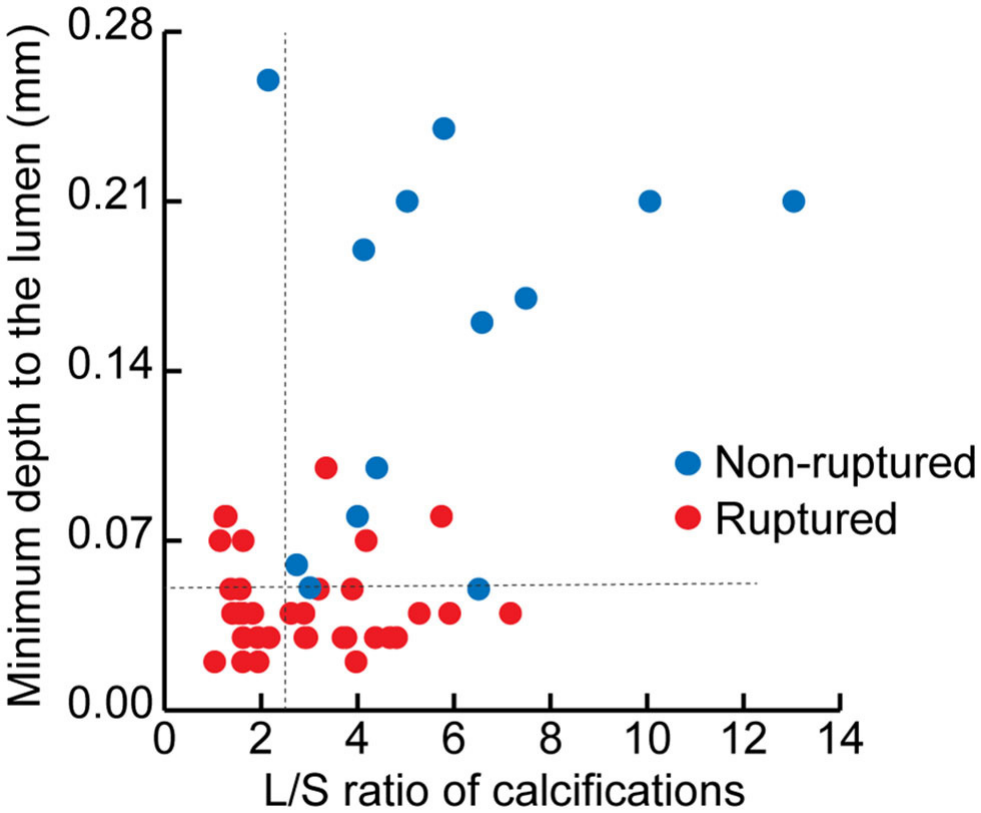
The distribution of calcifications in ruptured and non-ruptured plaques according to the minimal depth from the calcification to the lumen and the long axis/short axis (L/S) ratio of each calcification.

The depth from the calcification to the lumen was also measured by OCT. Remarkably, the distance to the lumen of non-ruptured plaques was more than four times the length of the distance in ruptured plaques (0.17 vs. 0.04 mm, *p* < 0.001). The potential cut-off values of minimal distance predicting plaque rupture were also measured (Supplementary Table V). We therefore defined calcifications with depth ≤ 50 μm as *superficial calcifications*, and defined calcifications with depth > 50 μm as *deep calcifications* (AUC, 0.94; 95% CI, 0.88–0.99; *p* < 0.001). Superficial calcifications accounted for 79.4% of all calcifications in the ruptured plaques, and only 7.7% in the non-ruptured plaques (*p* < 0.001). The distribution of calcification shape and location in the ruptured and non-ruptured plaques are shown in Figure 3. Superficial calcifications with rotund shape were mostly observed in ruptured plaques. Multivariable logistic analysis revealed that superficial calcification and rotund calcification were independently associated with plaque rupture.

Other geometric features of calcifications were also gauged. Calcifications in the ruptured and non-ruptured plaques had similar area size. However, calcifications in ruptured plaques had smaller circumference (4.67 vs. 6.43 mm, *p* = 0.049) and surface circumference/area ratio (3.92 vs. 6.16, *p* = 0.041). Such difference may be relevant to the calcification shape. Geometrically, the calcification with smaller L/S means rounder calcification with less surface circumference/area. Moreover, lesions with ≥50% stenosis also showed lower calcification area, circumference and calcification index, as well as lower surface circumference/area (Supplementary Table III). Besides, calcifications were located closer to the MLA site in ruptured plaques than in non-ruptured plaques (1.10 vs. 2.65 mm, *p* = 0.04). The longitudinal calcification length, calcification arc and calcification index were similar between ruptured and non-ruptured plaques. No difference was observed in the number of spotty calcifications between two groups. Nevertheless, ruptured plaques had more protruded calcification than non-ruptured plaques (47.1 vs. 0.0%, *p* = 0.002).

### Biomechanical Analysis

**Figure 4:**
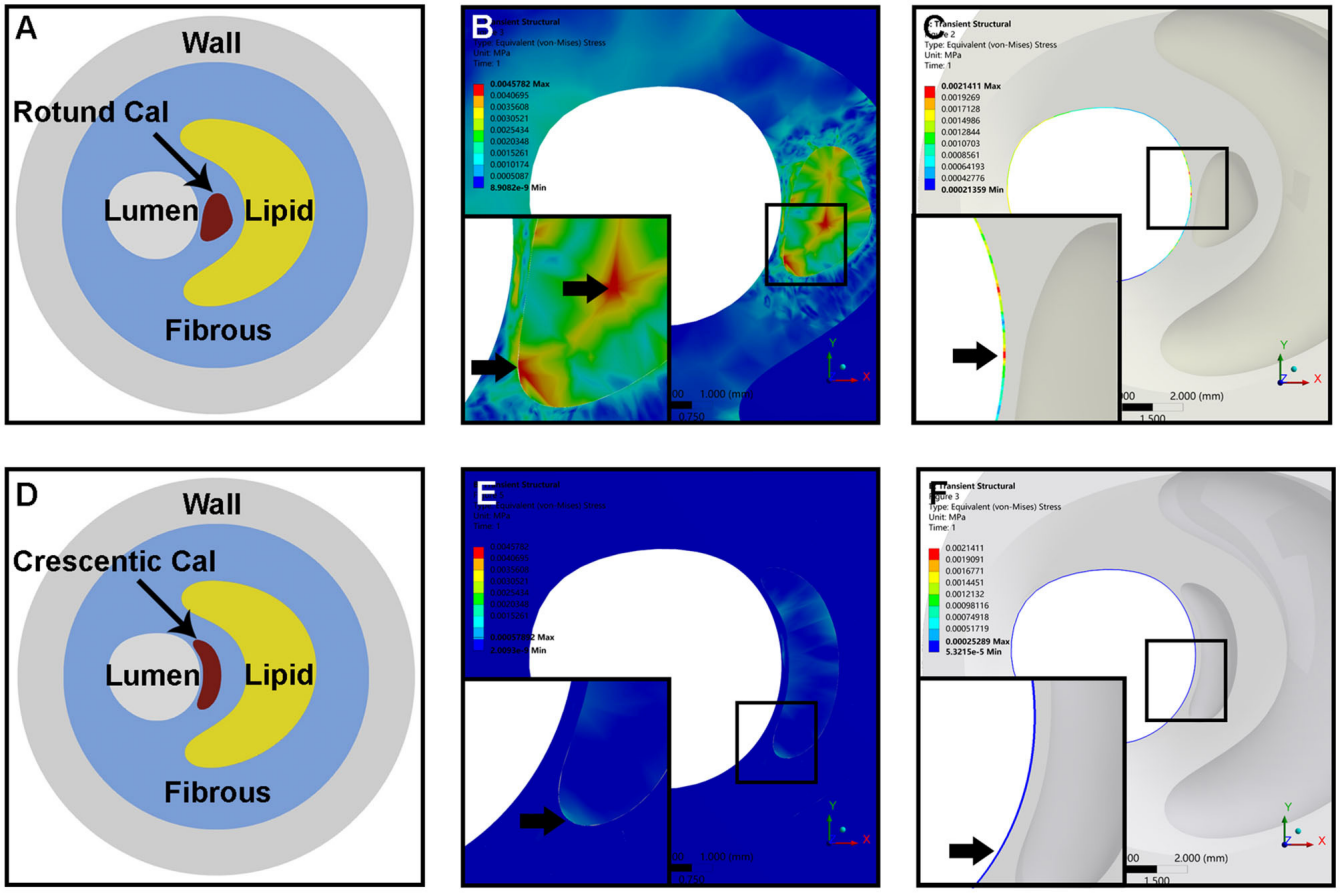
The idealized models of an atheromatous plaque with rotund calcification **(A)** and crescentic calcification **(D)**. Representative of von Mises stress distribution within the whole plaque **(B,E)** and the fibrous on the lumen surface **(C,F)** of rotund calcification and crescentic calcification; the arrow indicated the location of maximum von Mises stress within the whole plaque and separated fibrous tissue.

We subsequently constructed two idealized models of rotund and crescentic calcifications with same size and same distance to the lumen surface. FSI models were used to determine the influence of calcification shape on the plaque structural stress. Figure 4 displayed the von Mises stress distribution on the cross-sectional plane of maximum calcification area. The peak stress concentrated at the interface between calcification and fibrous on both calcification models. Maximum von Mises stress in the plaque with rotund calcification was 7.91-fold higher than that of the model with crescentic calcification (4578.2 vs. 578.92 Pa). Furthermore, calcification indirectly affect the stress on the remote lumen surface. Maximum von Mises stress on the fibrous on the lumen surface was 8.47-fold higher in the model with rotund calcification (2141.1 vs. 252.89 Pa). Furthermore, maximum flow shear stress on rotund calcification model was 7.94-fold greater than that on crescentic calcification model (2598.8 vs. 327.35 Pa).

## Discussion

The major findings of our investigation are as follows. (1) The frequency of calcification was similar between ruptured and non-ruptured plaques. Ruptured plaques had more multiple calcifications and protruded calcifications. (2) Superficial calcifications and rotund-shaped calcifications were independently associated with plaque rupture. (3) Rotund-shaped calcification exerted significantly higher von Mises stress on the plaque in the idealized FSI models.

Most previous studies differentiated between calcified and non-calcified plaques, spotty and large calcifications, single and multiple calcifications to determine the role of calcification in the plaque stability. With OCT, we detected a more detailed architecture of calcifications in the carotid plaque, including calcification shape, location, quantitative measurements of semilunar axis, area size, circumference, cross-sectional arc, longitudinal length, and etc. Since calcifications on cross-sectional planes are somewhat moon-shaped at different phases, we first classified calcifications into crescentic or rotund calcifications based on their L/S ratios. Rotund-shaped calcifications strongly associated with plaque rupture. Biomechanical analysis using idealized FSI models may provide an explanation for this association. Rotund-shaped calcifications exerted more stress on the calcification-fibrous boundary and the luminal surface.

Geometrically, the calcification with smaller L/S means rounder calcification with less circumference/area. Thus, a rotund calcification will have less fibrous interface than a crescentic calcification with same calcified contents (same area). Calcified plaque is at least 4 to 5 times more stiff than cellular plaque (22). Failure stress, which tends to occur at interfaces between materials of different stiffness, would concentrate at interfaces between calcified and non-calcified materials, such as fibrous and lipid core within a plaque (5, 23). In the atherosclerotic process, the mechanical instability introduced by the interfacing areas may promote plaque rupture and symptomatic events (23, 24). A computational study using idealized plaque models indicated calcifications shape may affect plaque stress (25). As shown in our idealized models, peak stress concentrated at interfaces between calcium and fibrous, especially in plaques with rotund calcification. The mechanical instability in plaque with rotund calcification, manifested as flow maximum shear stress and von Mises stress, increased much more than that in plaque with crescentic calcification. Calcification, on the other hand, is not quiescent but an active stage of atherosclerosis related to oxidized lipids and inflammatory activity (6, 7). We speculate that these rotund calcifications, particularly those superficial rotund calcifications, might be a form of early intimal calcifications, which develop in the fibrous cap and shoulder regions of complicated atherosclerotic lesions provoked by focal accumulations of inflammatory cells (26). The relationship of inflammatory cytokines from macrophages and calcification might be a positive feed-back loop (6), thereby promoting plaque vulnerability.

Another quantitative measurement we made in this investigation is the minimal distance from the calcification to the lumen. Plaque rupture was associated with a remarkable shortened depth, which equates to approximately one-fourth of that in the non-ruptured plaque. Superficial calcifications within 50 μm from the lumen appeared more frequently in the ruptured plaques. Several investigations have described the association between calcification location and the vulnerable plaque. However, there is a discrepancy in the determination of *superficial* calcifications. Superficial calcification was first introduced in coronary artery by means of intravascular ultrasound as calcification located at the intimal-lumen interface or closer to the lumen than to the adventitia (27). Two recent studies on carotid atherosclerotic plaque employed this definition to examine the association of calcification location and intraplaque hemorrhage. One used carotid CT angiography source images to illustrate superficial calcifications (28). The other defined surface calcifications as calcified nodule within or very close to the fibrous cap but without fibrous tissue completely covering by means of MRI (9). As for the OCT imaging, Jang's group utilized 65 or 100 μm criteria to define superficial calcium and found no connection between calcium location and symptomatic plaques (17). However, their subsequent investigation demonstrated sheet-like superficial calcific plate without erupted nodules or protruding mass into the lumen accounts for 67.4% of calcified plaques at the culprit site (19). Yu's group found that minimum depth of 63 μm or even less of calcification is the critical cutoff point for coronary lipid-rich calcified plaque rupture (sensitivity = 77.8%, specificity = 81.8%) (29). In a similar way of ROC curve analysis, we detected 50 μm is the cutoff point of superficial calcifications with 79.4% sensitivity and 84.6% specificity for carotid plaque rupture.

The mechanism of how superficial calcification stimulates plaque rupture is unclear. When failure stress concentrate at interfaces, superficial calcifications lead to less buffer force provided from the fibrous tissue. Superficial calcifications increase local stress and stretch concentration on the plaque surface (30). Pathologically, the inflammatory cell, such as macrophages or mast cells, may perpetuate the inflammatory response and then increase the risk of plaque destabilization and local thrombosis (26). In contrast, deep calcifications are usually located near the adventitia, which serves as a barrier restraining the spread of inflammatory substance (31).

We further investigated that larger calcification area, lower calcification surface and lower circumference/area ratio were indicated in lesions with ≥50% stenosis. This was consistent with previous findings that small calcifications were associated with more extensive atherosclerosis and accelerated disease progression (7). Moreover, lesions with severe stenosis presented more frequently with TCFA than lesions with mild stenosis, whereas no significant difference was showed in the incidence of plaque rupture, thrombus and macrophage accumulations. Previous histopathological studies have indicated that a thin or ruptured fibrous cap, a large lipid-rich core and macrophage accumulations could also occur in plaques that do not cause significant luminal stenosis (32). Most accelerated coronary plaque lesions which had causing ischemic symptoms were also visualized as only mildly or moderately stenotic (33). Despite only mild or moderate stenosis of ICA on angiographic imaging was indicated, these plaques may still have a high risk of rupture and embolization in some cases (34, 35).

This study has several limitations. First, OCT might miss some calcifications distal from the lumen, notably behind a large lipid core or intraluminal thrombus. The deep calcifications, therefore, may be underestimated. Second, the study population was small, including 63 carotid plaques totally. Each patient whether underwent OCT imaging or not was at the discretion of each operator. Therefore, selection bias cannot be excluded. Most of the plaques for OCT scan were from carotid stenosed within 30–70%. Only 3.17% of plaques (2/63) were evaluated from severe stenosis (>70%), which has been reported to linked with severe calcifications located the outside layer of the atheroma (36). The less inclusion of severe carotid stenosis may lead to less calcified plaques, particularly those deep calcifications. Third, we investigated the calcification shape in the cross-sectional OCT frames. The three-dimensional geometry of calcification still awaits further studies.

## Conclusion

Superficial calcifications with rotund shape in carotid atherosclerotic plaques are associated with plaque rupture, suggesting that calcification shape and location may play a key role in plaque vulnerability.

## Data Availability Statement

The raw data supporting the conclusions of this article will be made available by the authors, without undue reservation.

## Ethics Statement

The studies involving human participants were reviewed and approved by the Ethics Committee of Jinling Hospital. The patients/participants provided their written informed consent to participate in this study.

## Author Contributions

XS: study design, OCT image collection and analysis, statistical analysis, and manuscript drafting. YH and RL: OCT image collection and analysis. ML: image collection and interpretation of results. QY, FW, and XX: data collection. YX: critical revision of manuscript. RY: study design, interpretation of results, and critical revision of manuscript. XL: study design. All authors contributed to the article and approved the submitted version.

## Conflict of Interest

The authors declare that the research was conducted in the absence of any commercial or financial relationships that could be construed as a potential conflict of interest.
