# Supplementary material for: Superficial Calcification With Rotund Shape Is Associated With Carotid Plaque Rupture: An Optical Coherence Tomography Study
Source: Front Neurol. 2020 Sep 18;11:563334. doi: 10.3389/fneur.2020.563334 (PMC7530839; doi:10.3389/fneur.2020.563334)
Supplement: Supplementary file 1 [file Table_1.pdf]

Superficial Calcification with Rotund Shape is Associated with Carotid Plaque Rupture: An Optical Coherence Tomography Study

Xuan Shi[1], MD, Yunfei Han[1], MD, PhD, Min Li[2], MD, Qin Yin[1], MD, Rui Liu[1], MD, PhD, Fang Wang[1], MD, Xiaohui Xu[1], MD, Yunyun Xiong[3,4], MD, PhD, Ruidong Ye[1*], MD, PhD, Xinfeng Liu[1*], MD, PhD

[1] *Department of Neurology, Affiliated Jinling Hospital, Medical School of Nanjing University, Nanjing, China*

[2]*Department of Neurology, Jiangsu Province Hospital of Chinese Medicine, Nanjing University of Chinese Medicine, Nanjing, China*

[3] *China National Clinical Research Center for Neurological Diseases, Beijing, China,*

[4] *Vascular Neurology, Department of Neurology, Beijing Tiantan Hospital, Capital Medical University, Beijing, China*

[*]*Correspondence to: R. Ye (Email: yeruid@gmail.com; Tel: 86-25-80860454; Fax: 86-29-83251302) and X. Liu (Email: xfliu2@vip.163.com; Tel: 86-25-80863485; Fax: 86-25-84664563), Department of Neurology, Affiliated Jinling Hospital, Medical School of Nanjing University, 305 East Zhongshan Rd, Nanjing, 210002, China*

SUPPLEMENTARY MATERIAL

Table of Contents

Supplemental Methods. OCT Image Analysis
Supplemental Figure. Representative optical coherence tomography images of rupture plaque and non-rupture plaque
Supplemental Table I. OCT Analysis of Plaque Characteristics in Ruptured and Non-Ruptured Plaques
Supplemental Table II. OCT Analysis of Plaque Characteristics in Lesions With < 50% and ≥ 50% Stenosis
Supplemental Table III. Calcification Characteristics in Lesions With < 50% and ≥ 50% Stenosis
Supplemental Table IV. Sensitivity, specificity, LR −, and LR + of different cut points of the long-axis/short-axis ratio for predicting plaque rupture
Supplemental Table V. Sensitivity, specificity, LR −, and LR + of different cut points of the distance between calcification and the lumen for predicting plaque rupture

Supplemental Methods

OCT Image Analysis

To determine the reproducibility of OCT qualitative assessments, data were analyzed by 2 independent analysts (X.S. and R.L.) and repeated 1 month after initial analysis by the same analyst (X.S.). We demonstrated inter-observer variability for identification of plaque rupture (κ = 0.96), total calcification deposits (κ = 0.85), and rotund calcification (κ = 0.88). The measurement of the minimum depth from calcification to the luminal surface also showed good inter-observer reproducibility (intra-class correlation coefficient = 0.996). Intra-observer agreement, evaluated twice by one observer within one-month interval, showed excellent reproducibility (plaque rupture, κ = 0.96; total calcification deposits, κ = 0.94; rotund calcification, κ = 0.94; the minimum depth from each calcification to the luminal surface, intra-class correlation coefficient = 0.997).

Supplemental Figure

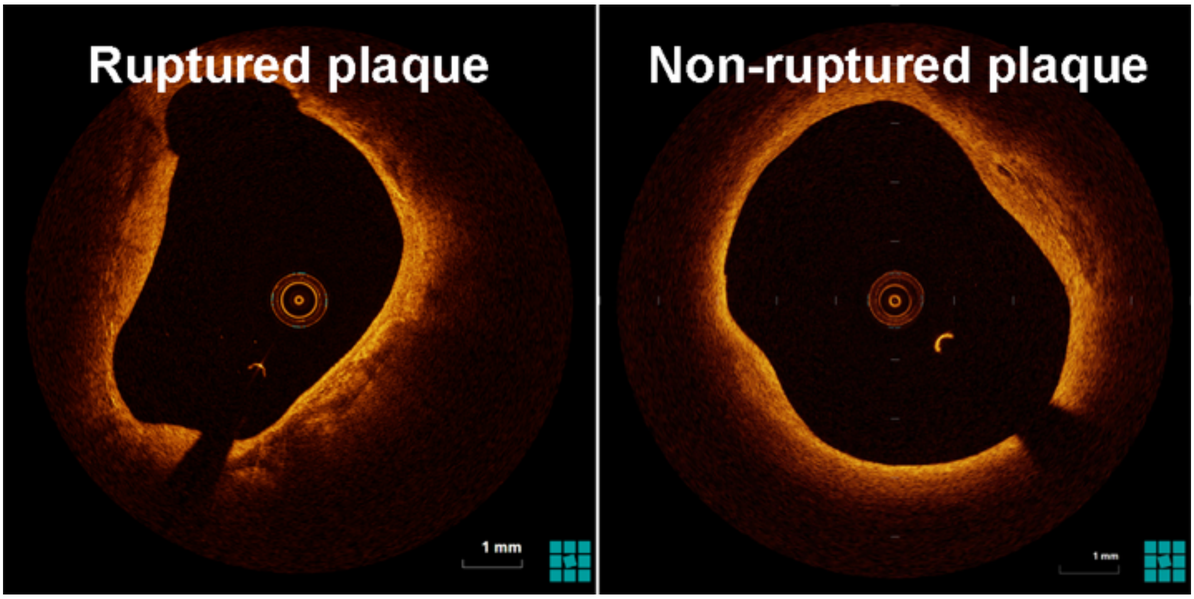

Representative optical coherence tomography (OCT) images of rupture plaque and non-rupture plaque.

Supplemental Table I.

OCT Analysis of Plaque Characteristics in Ruptured and Non-Ruptured Plaques

	Ruptured plaques (n = 33)	Non-ruptured plaques (n = 30)	*p* value
MLD, mm	3.10 ± 1.09	3.63 ± 0.78	0.03
MLA, mm^2	7.34 (3.95-13.22)	10.50 (6.61-14.64)	0.04
RVD, mm	4.87 (4.16-5.59)	5.06 (4.42-5.97)	0.41
RVA, mm^2	19.41 ± 7.99	21.79 ± 8.60	0.27
DS, %	37.94 ± 19.52	30.38 ± 14.38	0.11
AS, %	55.96 ± 22.83	49.34 ± 20.51	0.26
Lipid-rich plaque, n (%)	27 (81.82)	12 (40.00)	0.001
TCFA, n (%)	25 (75.76)	7 (23.33)	< 0.001
Minimum FCT, mm	0.05 (0.03-0.06)	0.07 (0.05-0.10)	< 0.001
Thrombus, n (%)	9 (27.27)	0 (0.00)	0.002
Macrophages, n (%)	20 (60.61)	12 (40.00)	0.13
Cholesterol crystals, n (%)	9 (27.27)	5 (16.67)	0.37
Neovascularization, n (%)	6 (18.18)	0 (0.00)	0.02
Calcification, n (%)	15 (45.45)	9 (30.00)	0.30
Multiple calcifications, n (%)	11 (33.33)	3 (10.00)	0.04

Continuous variables were presented as mean ± SD or median with 25th to 75th percentile. Categorical data were expressed as frequency and percentage. MLD, minimum luminal diameter; MLA, minimum luminal area; RVD, reference vessel diameter; RVA, reference vessel area; AS, area stenosis; DS, diameter stenosis; TCFA, thin-capped fibroatheroma; FCT, fibrous cap thickness.

Supplemental Table II.

OCT Analysis of Plaque Characteristics in Lesions With < 50% and ≥ 50% Stenosis

	Plaques with < 50% stenosis (n = 52)	Plaques with ≥ 50% stenosis (n = 11)	*p* value
Lipid-rich plaque, n (%)	29 (55.77)	10 (90.91)	0.04
TCFA, n (%)	23 (44.23)	9 (81.82)	0.04
Minimum FCT, mm	0.05 (0.03-0.06)	0.07 (0.05-0.10)	0.06
Plaque rupture, n (%)	26 (50.00)	7 (63.63)	0.52
Thrombus, n (%)	6 (11.54)	3 (27.27)	0.18
Macrophages, n (%)	25 (48.08)	7 (63.63)	0.51
Cholesterol crystals, n (%)	10 (19.23)	4 (36.36)	0.24
Neovascularization, n (%)	4 (7.69)	2 (18.18)	0.28
Calcification, n (%)	18 (34.62)	6 (54.55)	0.31
Multiple calcifications	10 (19.23)	4 (36.36)	0.24

Continuous variables were presented as mean ± SD or median with 25th to 75th percentile. Categorical data were expressed as frequency and percentage. TCFA, thin-capped fibroatheroma; FCT, fibrous cap thickness.

Supplemental Table III. Calcification Characteristics in Lesions With < 50% and ≥ 50% Stenosis

	Calcifications in lesions with < 50% stenosis (n = 35)	Calcifications in lesions with ≥ 50% Stenosis (n = 12)	*p* value
L/S	3.35 (1.83-5.28)	2.16 (1.58-4.32)	0.23
Rotund calcification, n (%)	10 (28.57)	7 (58.33)	0.06
Crescentic calcification, n (%)	25 (71.43)	5 (41.67)	0.06
Minimal distance to the lumen, mm	0.05 (0.04-0.08)	0.04 (0.03-0.16)	0.58
Superficial calcification, n (%)	23 (65.71)	7 (58.33)	0.73
Deep calcification, n (%)	12 (34.29)	5 (41.67)	0.73
Calcification area, mm^2	1.50 (0.69-2.68)	0.50 (0.29-1.09)	0.002
Calcification surface, mm	5.73 ± 2.82	3.47 ± 1.81	0.013
Surface circumference/area, mm/mm^2	3.63 (2.78-5.60)	6.41 (3.96-7.10)	0.03
Calcification length, mm	2.48 ± 1.62	1.52 ± 1.08	0.06
Maximum calcification arc, °	41.65 (23.38-54.18)	49.30 (38.10-77.30)	0.12
Mean calcification arc, °	42.58 (31.80-61.17)	34.35 (23.38-50.80)	0.29
Calcification index, °mm	86.64 (39.44-194.58)	41.27 (19.67-90.74)	0.04
Spotty calcification, n (%)	28 (80.00)	12 (100.00)	0.17
Protruded calcification, n (%)	12 (34.29)	4 (33.33)	1.00

L/S, long axis/short axis ratio; MLA, minimum luminal area.

Supplemental Table IV.

Sensitivity, specificity, LR −, and LR + of different cut points of the long-axis/short-axis ratio for predicting plaque rupture

Cut points	Sensitivity (95% CI)	Specificity (95% CI)	LR + (95% CI)	LR - (95% CI)
≤ 1.5	17.65 (7.4-35.2)	100.00 (71.7-100.0)	-	0.82 (0.7-1.0)
≤ 2.0	44.12 (27.6-61.9)	100.00 (71.7-100.0)	-	0.56 (0.4-0.8)
≤ 2.5	47.06 (30.2-64.6)	92.31 (62.1-99.6)	6.12 (0.9-41.6)	0.57 (0.4-0.8)
≤ 3.0	58.82 (40.8-74.9)	76.92 (46.0-93.8)	2.55 (0.9-7.2)	0.54 (0.3-0.8)
≤ 3.5	64.71 (46.5-79.7)	76.92 (46.0-93.8)	2.80 (1.0-7.8)	0.46 (0.3-0.8)
≤ 4.0	76.47 (58.4-88.6)	69.23 (38.9-89.6)	2.49 (1.1-5.7)	0.34 (0.2-0.7)
≤ 5.0	88.24 (71.6-96.2)	53.85 (26.1-79.6)	1.91 (1.0-3.5)	0.22 (0.1-0.6)
≤ 6.0	97.06 (82.9-99.8)	38.46 (15.1-67.7)	1.58 (1.0-2.4)	0.08 (0.01-0.7)

LR −, negative likelihood ratio; LR +, positive likelihood ratio; CI, confidence interval

Supplemental Table V.

Sensitivity, specificity, LR −, and LR + of different cut points of the distance between calcification and the lumen for predicting plaque rupture

Cut points	Sensitivity (95% CI)	Specificity (95% CI)	LR + (95% CI)	LR - (95% CI)
< 0.02	0 (0.0 - 10.3)	100 (75.3 - 100.0)	-	1 (1.0 - 1.0)
≤ 0.04	67.65 (49.5 - 82.6)	100 (75.3 - 100.0)	-	0.32 (0.2 - 0.5)
≤ 0.05	79.41 (62.1 - 91.3)	84.62 (54.6 - 98.1)	5.16 (1.4 - 18.7)	0.24 (0.1 - 0.5)
≤ 0.06	79.41 (62.1 - 91.3)	76.92 (46.2 - 95.0)	3.44 (1.3 - 9.4)	0.27 (0.1 - 0.6)
≤ 0.07	88.24 (72.5 - 96.7)	76.92 (46.2 - 95.0)	3.82 (1.4 - 10.4)	0.15 (0.06 - 0.4)
≤ 0.08	97.06 (84.7 - 99.9)	69.23 (38.6 - 90.9)	3.15 (1.4 - 7.1)	0.042 (0.006 - 0.3)
≤ 0.1	100 (89.7 - 100.0)	61.54 (31.6 - 86.1)	2.6 (1.3 - 5.2)	-

LR −, negative likelihood ratio; LR +, positive likelihood ratio; CI, confidence interval
